# Retraction: Fahr Disease: A Rare Cause of First-Time Seizure in the Emergency Department

**DOI:** 10.7759/cureus.r104

**Published:** 2024-01-25

**Authors:** Faleh M Aldawsari, Turki B Alotaibi, Omran S Hashim, Zainab A Bu Hamad, Maha R Eisaa, Abdulrahman A Alhumaidi, Salem M Alanazi, Fahad F Alenezi, Afnan A Batwie, Abdulaziz A Habib, Sukainah S Alismail, Omar S Almulhim, Abdullah F Al Amer, Thamer A Alghamdi, Faisal Al-Hawaj

**Affiliations:** 1 General Practice, ​King Khalid Hospital, Al-Kharj, SAU; 2 Medicine, University of Debrecen, Debrecen, HUN; 3 National Guard Health Affairs, Prince Mohammed Bin Abdulaziz Hospital, Medina, SAU; 4 Medicine, Medical University of Warsaw, Warsaw, POL; 5 Medicine, Sulaiman Al Rajhi University, Qassim, SAU; 6 Medicine, King Saud bin Abdulaziz University for Health Sciences, Riyadh, SAU; 7 Medicine, University of Jordan, Amman, JOR; 8 Medicine, Jordan University of Science and Technology, Irbid, JOR; 9 Medicine, King Abdulaziz University, Jeddah, SAU; 10 General Practice, King Faisal Hospital, Al-Ahsa, SAU; 11 Medicine, King Faisal University, Hofuf, SAU; 12 Medicine, Gulf Medical University, Ajman, ARE; 13 Medicine, Al-Baha University, Al Baha, SAU; 14 Medicine, Imam Abdulrahman Bin Faisal University, Dammam, SAU

The Editors-in-Chief have retracted this article. Concerns were raised regarding the identity of the authors on this article. Specifically, Faisal Alhaway and Malak Shammari have stated that they were added to this article without their knowledge or approval. The identity of the other authors could also not be verified. As the appropriate authorship of this work cannot be established, the Editors-in-Chief no longer have confidence in the results and conclusions of this article.

